# Influence of Bone Density and Guide Protocol on the Accuracy of Self‐Cutting Implants Using Static Guided Implant Placement—An In Vitro Study

**DOI:** 10.1111/clr.14470

**Published:** 2025-07-03

**Authors:** Caroline Streichfuss, Stefan Wolfart, Lukas Waltenberger

**Affiliations:** ^1^ Department of Prosthodontics and Biomaterials, Centre for Implantology RWTH Aachen University Hospital Aachen Germany

**Keywords:** accuracy, bone density, computer‐aided, guide protocol, implant design, implant surgery, self‐cutting

## Abstract

**Introduction:**

To investigate the influence of bone density and guide protocol on the accuracy of static guided implant placement of self‐cutting implants in vitro.

**Methods:**

A total of 242 implant replicas of self‐cutting implants and implants with a tapered design as a control were placed in 40 maxilla replica models with healed posterior ridges differing in bone density and anterior extraction sockets. After full‐guided osteotomy, the implants in the healed ridge were either placed through the template or freehand. In anterior extraction sockets, a pilot‐guided group was additionally investigated. Postoperative scans were superimposed with the planning and investigated for angular, coronal, and apical deviation.

**Results:**

For 3D coronal deviation, the study indicates significantly lower deviations in soft bone (*p* < 0.01) and for guided implant placement (*p* < 0.01) in posterior sites. Apical and angular deviations are significantly influenced by the placement protocol (*p* ≤ 0.03) and the presence of reference points from neighbouring teeth (*p* < 0.01). In the anterior site, the coronal deviation seems to be improved by a direct view of the surgical field, while 3D apical and angular accuracy appears to benefit from a high degree of guidance (*p* < 0.01).

**Conclusion:**

Within the limitations of this in vitro study, for low bone density, immediate implant placement, and in clinically challenging situations without reference to the adjacent teeth, the use of template guidance for insertion statistically significantly reduced deviation.

## 1 Introduction

Static Computer Assisted Implant Surgery (sCAIS) is a routine treatment procedure that has been widely investigated in in vitro studies as well as in clinical studies for established protocols and implant geometries (Tattan et al. 2020). It promises a high degree of accuracy by precisely transferring the implant planning based on a three‐dimensional radiograph and surface scan to the subsequent implant positioning. The risk of complications, such as damage to neighbouring structures (e.g., nerves or teeth) or opening of the maxillary sinus, is minimized by targeted implant positioning. The existing bone can also be optimally utilized and augmentation procedures avoided or precisely planned. In addition, backward planning allows the subsequent position of the implants to be optimally aligned with the planned prosthetic restoration and increases the predictability of the subsequent treatment outcome and aesthetics (Gianfreda et al. 2022).

The available literature shows that even seemingly minor factors can affect the accuracy of static guided implantation. These relate to the variety of factors, such as jaw type, which tends to be more accurate in the maxilla (Pozzi et al. 2016; Tahmaseb et al. 2018). Further, the degree of edentulism affects accuracy, with tooth‐supported templates being superior to mucosa‐ and bone‐supported templates. Higher accuracy is seen in the gaps compared to free‐end situations (Raico Gallardo et al. 2017) and in situations where flap preparation is not required (Tahmaseb et al. 2018; Tattan et al. 2020). The chosen workflow, the type of imaging (Pozzi et al. 2016; Wismeijer et al. 2018) and surface capture (Gottlow and Sennerby 2024; Tahmaseb et al. 2018) as well as the evaluation strategy also have an impact on accuracy rating (Lin, Wu, et al. 2020; Skjerven et al. 2019; Varga Jr. et al. 2020). Even the influence of the design of the drilling template, with higher accuracy for closed sleeves compared to open sleeve design, was shown. Large sleeve‐guided surfaces, that is, length of the guide through the drill tray key height and sleeve size with a small distance above the sleeve (free drilling distance) offered advantages in terms of accuracy (El Kholy, Janner, et al. 2019; El Kholy, Lazarin, et al. 2019). An ideal tooth to template distance (offset) should be set for an optimal template fit (Lim et al. 2021) and the influence of the implant geometry, with less deviation for conical compared to straight implants (El Kholy, Ebenezer, et al. 2019), was analyzed in further studies.

The sCAIS is subdivided according to different protocols that determine the degree of guidance of the drilling template during osteotomy and implant placement. A recent meta‐analysis showed that sCAIS is associated with superior accuracy compared to conventional freehand implant placement, except in scenarios where pilot drill guidance is used alone (Mahardawi et al. 2025). The included studies varied considerably in terms of implant location and the extent of edentulism.

New implant systems use more aggressive self‐cutting thread geometries. These systems aim to achieve a predictably higher insertion torque by using underdrilling, that is, an osteotomy of the implant site with a smaller diameter than the external geometry of the implant (Herrero‐Climent et al. 2020; Yu et al. 2022). The increased primary stability achieved in this way enables the integration of these systems into direct protocols, as already known for the immediate restoration of individual posterior regions with a prefabricated customized abutment (Waltenberger et al. 2024), or the immediate restoration in the edentulous jaw (Norré and Att 2022).

A prerequisite for the application is a high predictability of the implant position. The more aggressive thread design may result in greater deviations, as the cutting thread flanks already determine the initial position of the implant, whereas non‐cutting and tapered implants may initially slide more passively into the bone after the osteotomy. In addition, different initial clinical situations pose challenges: The standard situation considered is (1) late implantation in the posterior area with varying bone density, (2) the tight alveolar crest with little cortical bone, and lastly (3) immediate implant placement in the anterior area where there is often a strong palatal cortical bone and the alveolus itself offers little resistance to osteotomy and implant placement.

The aim of the present study was to investigate the accuracy of self‐cutting implants with statically guided implant osteotomy and different insertion protocols in the clinical situations mentioned above.

The null hypotheses are that bone density, insertion protocol, presence of adjacent teeth for reference in late implant placement, and surgeon experience in immediate anterior implant placement have no effect on implant position accuracy.

## 2 Materials and Methods

### 2.1 Bone Model and Implants

This study was conducted in vitro on acrylic models (U‐026A, U‐026B, Bonemodels, Castellon, Spain) in order to simulate standardized bone densities of a human maxilla. The eligibility of the acrylic models for the assessment of implant accuracy was previously demonstrated in large preclinical studies (El Kholy, Janner, et al. 2019; El Kholy, Lazarin, et al. 2019).

**FIGURE 1:**
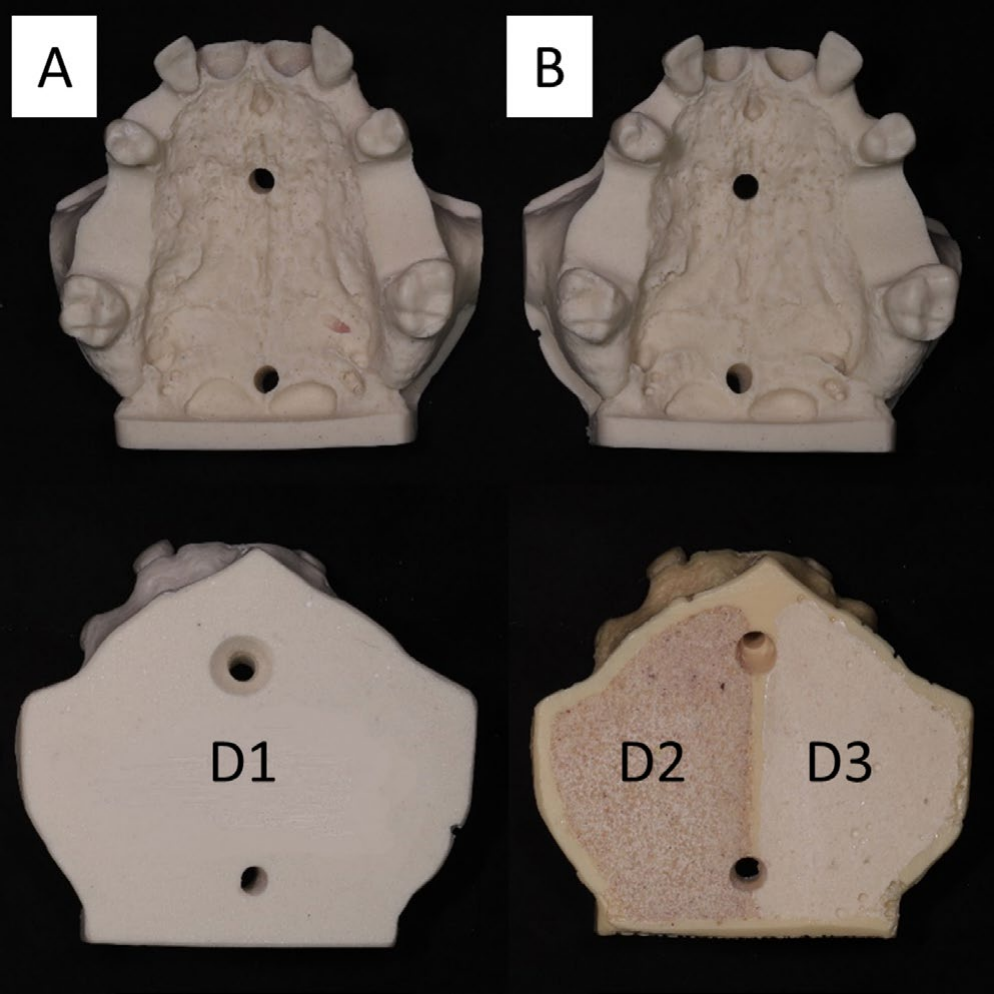
Replica models with healed posterior ridges differing in bone density and anterior extraction sockets. (A) Bonemodel with bone density D1 in posterior sites. (B) Bonemodel with bone density D2 and D3 in posterior sites.

The models provided eight implant insertion sites following a standardised modification protocol with bone reduction in the first molar positions to ensure a consisted cortical thickness of 2 mm (±0.5 mm). The bone morphology simulated a healed bone crest in FDI positions 16, 15, 25, and 26. Model types A and B differentiated between bone densities in posterior implant position of D1, D2 and D3 according to Lekholm and Zarb. FDI positions 13 and 23 simulated a narrow and tilted ridge with limited visual guidance for implant placement from adjacent teeth. FDI positions 11 and 21 simulated immediate extraction sockets (Figure 1).

No artificial gingiva was added to the models.

**FIGURE 2:**
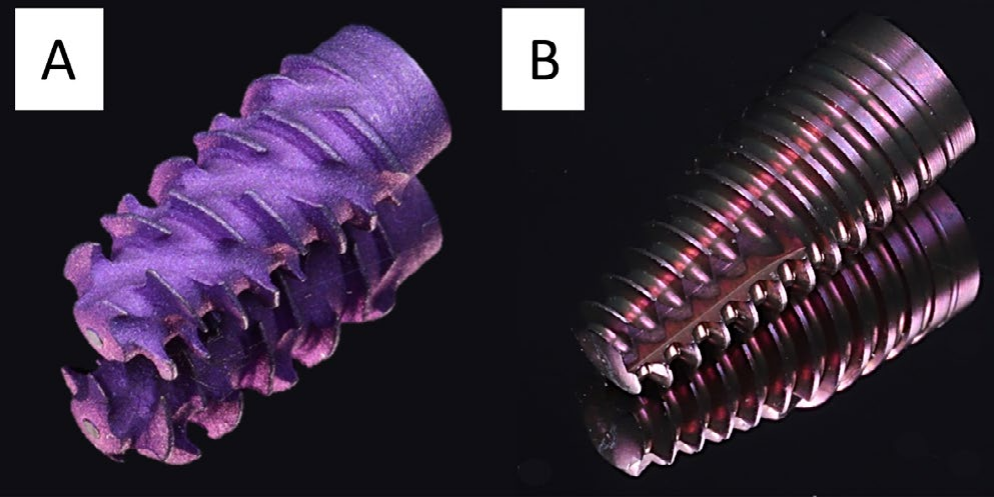
Original implant replicas used in the study. (A) Test group: Self‐cutting implant (Bone Level X Implant, Straumann AG). (B) Control group: Cylindrical tapered implant (Bone Level Tapered Implant, Straumann AG).

The assessed implant type (Bone Level X Straumann AG, Basel, Switzerland) is characterized by an aggressive macro design: wide interrupted helical threads and a narrower implant body with a reverse tapered implant neck. The macro design allowed a drilling protocol to be adjusted for bone density, allowing underdrilling to increase primary stability in softer bone. As a control, a tapered cylindrical implant with a strict drilling sequence was selected (Bone Level Tapered, Straumann AG). Original implant replicas with identical macro design were used throughout the study (Figure 2).

### 2.2 Virtual Implant Planning

To account for slight variation in the dimensions of the models, a Cone Beam Computer Tomography (CBCT, Orthophos SL, Sirona Dental Systems GmbH, Bensheim, Germany) with a voxel size of 0.16 × 0.08 mm and a three‐dimensional optical surface laboratory scan (Medit T710, Medit Corp, Seoul, Korea) were performed for each specimen separately and imported into a virtual implant planning software (coDiagnostiX, version 10.4, Dental Wings, Montreal, Canada). After segmentation of the CBCT, the surface scan was superimposed using the remaining teeth as a reference, followed by visual verification of the fit in all slices (axial, coronal, sagittal).

**FIGURE 3:**
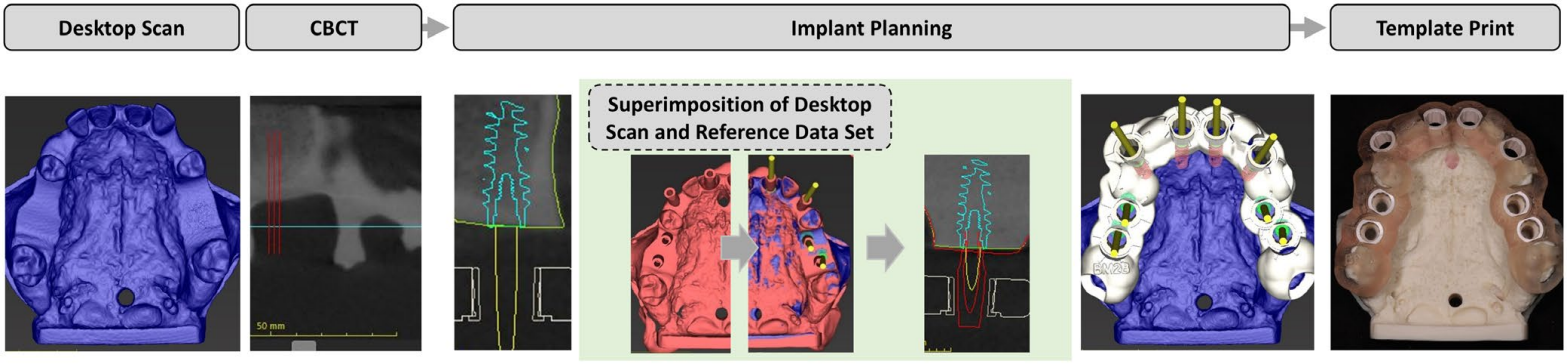
Digital Workflow—Implant planning based on a CBCT and a desktop scan and superposition of reference data set (STL, red) with data set of desktop scan of planning model (STL, blue); subsequent designing of the template and template print.

In order to achieve comparable planning for all models, a virtual master planning was exported with scan bodies as references (STL data set). This master reference was superimposed on all the other models to ensure consistent planning. Implant positions were only fine‐tuned according to the bone contour (Figure 3). A minimum distance of 3.0 mm between the implants and 1.5 mm between the implant and the adjacent tooth was maintained (Gastaldo et al. 2004; Tarnow et al. 2000). For the posterior sites (FDI position 14, 16, 24, 26), the implant positions were planned at bone level in the center of the alveolar ridge. Implants were aligned parallel to each other and to adjacent teeth. In the canine region (FDI 13, 23), due to the narrow alveolar ridge, the implant position was planned according to the bone contour and at bone level. For anterior positions (FDI position 11, 21) the implant position was determined according to the criteria of Buser et al. (2004). A parallel alignment of the implants was ensured. The implant apex was aligned palatally, and the implant shoulder was positioned at the level of the palatal lamella.

The remaining teeth allowed the construction of strictly tooth‐supported guides. According to the manufacturer's guidelines, polyetheretherketone sleeves for the test groups and stainless‐steel sleeves in the control groups with a height of 5 mm were selected. The distance from the sleeve to the implant shoulder was set uniformly at 4 mm for all guides. In the construction, an offset of 0.2 mm, a wall thickness of 3.0 mm, and a large connector thickness were set equally. The virtually designed templates were exported as an STL file and manufactured by a stereolithographic printer (Formlabs, Form 2, Berlin, Germany) using surgery‐grade resin (Surgical Guide Resin, Formlabs). The templates were 3D printed, washed, and polymerized (Form 2; Form Wash, Form Cure, Formlabs GmbH, Berlin, Germany). Prior to drilling, all templates were checked for a tension‐free and rotation‐proof fit.

To ensure consistency during the study, all posterior procedures were performed by the same trained surgeon (C.S.) The training protocol included 100 implant osteotomies and placements in equal bone models in all bone densities. As implant placement in the anterior maxilla is particularly technique‐sensitive, drilling and placement in extraction sockets throughout all three subgroups were performed by a highly experienced surgeon (S.W.).

### 2.3 Study Groups

The following three insertion protocols were distinguished in this study: (1) fully guided osteotomy and implant placement (*Insert_guided*), (2) fully guided osteotomy, freehand implant placement (*Insert_free*), (3) fully guided pilot drilling, freehand implant placement (*Pilot only_Insert free*).

#### 2.3.1 Placement in Posterior Sites With Assessment of Bone Density and Insertion Technique

Preliminary testing determined the appropriate osteotomy protocol for each bone density, with a desired insertion torque of 50–60 Ncm. This torque level proved to be consistent in preliminary testing, allowing the drilling sequences to be adapted to bone densities with different levels of underdrilling. Underdrilling was performed for bone densities D2 and D3. The selected drilling sequence in relation to bone density is shown in Table 1.

**TABLE 1:** The selected drilling sequence for guided osteotomy and achieved insertion torque in relation to bone density for late implant placement.

Bone density	Drilling sequence determined in preliminary testing					Insertion torque (in Ncm)
Guided osteotomy (in cm)	Ø2.2	Ø2.8	Ø3.5	Ø3.7	Ø4.2	
BLX 4.5 × 12 mm						
D1	x	x		x	x	55
D2	x	x		x	Cortical (6 mm)	55
D3	x	x		x	Cortical (6 mm)	50
BLX 3.75 × 10 mm						
D1	x		x	Cortical (6 mm)		50

A total of 120 implants with a length of 12 mm and a diameter of 4.5 mm (BLX, Straumann AG) were inserted in different bone densities D1, D2, and D3 and insertion protocols (*Insert_guided* and *Insert*_*free*).

**FIGURE 4:**
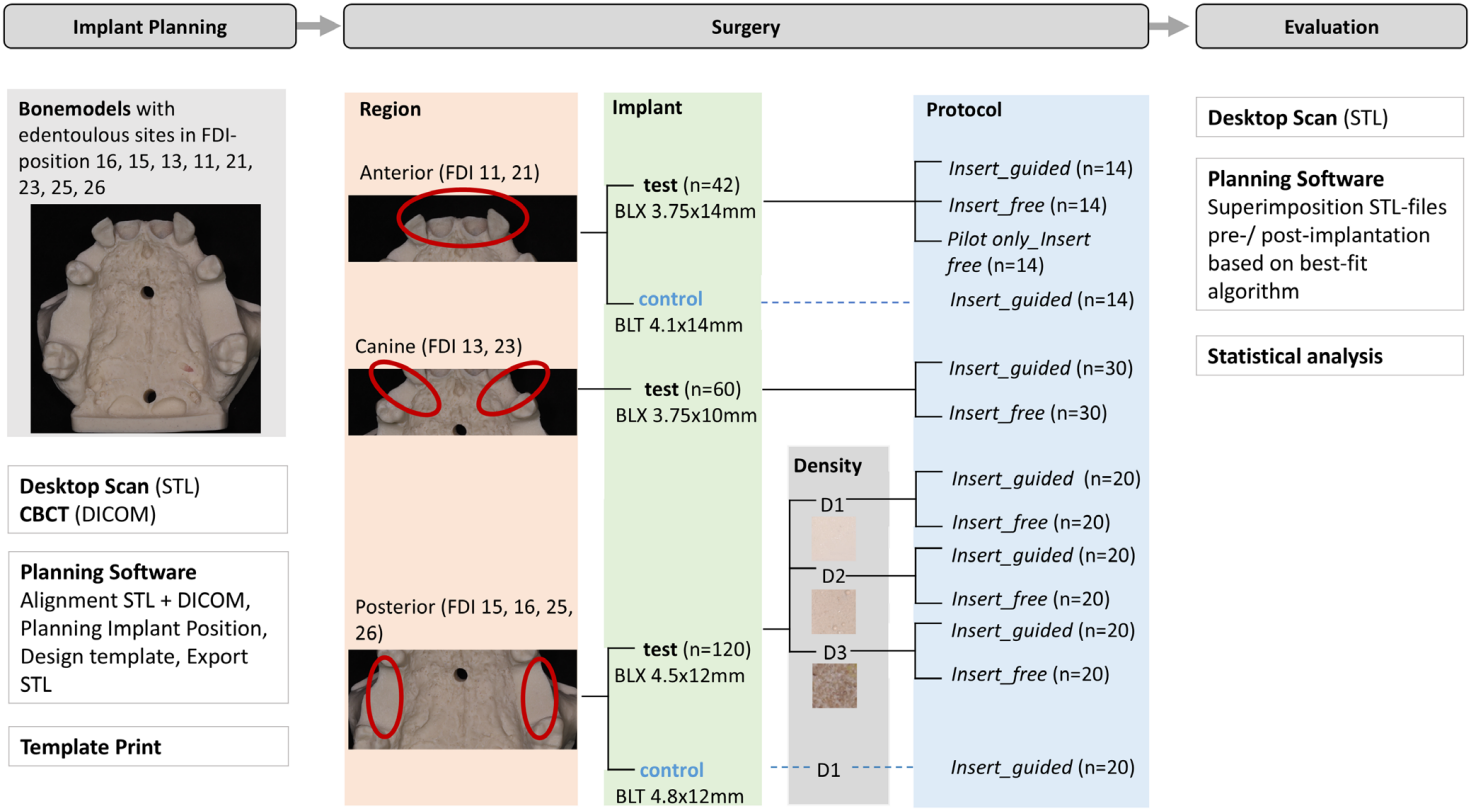
Study flow chart.

As control, 20 tapered straight implants with a length of 12 mm and a diameter of 4.8 mm (BLT, Straumann AG) were placed *Insert_guided* in bone density D1 (control) (Figure 4).

#### 2.3.2 Placement in Narrow Angulated Ridges With Different Insertion Techniques

A total of 60 implants with a length of 10 mm and a diameter of 3.75 mm (BLX, Straumann AG) were inserted in the canine region in bone density D1 using the insertion protocols *Insert_guided* and *Insert_free* (Figure 4).

#### 2.3.3 Immediate Anterior Placement With Different Drilling Protocols, Operator Experience and Insertion Technique

A total of 42 implants with a length of 14 mm and a diameter of 3.75 mm (BLX, Straumann AG) were inserted in simulated extraction sockets in FDI position 11 and 21 using the insertion protocols *Insert_guided*, *Insert_free*, and *Pilot only_Insert free*. In the *Pilot only_Insert free* protocol, only the 2.2 mm pilot drill was used fully guided through the template.

As control, 14 tapered straight implants with a length of 14 mm and a diameter of 4.1 mm were placed *Insert‐guided* (BLT, Straumann AG) (Figure 4).

### 2.4 Assessment of Implant Accuracy

A laboratory scan of the three‐dimensional surface of all models (Medit T710, Medit Corp) including inserted implants and the corresponding implant scanbodies (CARES mono‐RB, Straumann AG) was performed. The corresponding data set was exported in STL format. With the use of the treatment evaluation tool (coDiagnostiX), the planning software detected the three‐dimensional surface of the scanbody, and the implant position was defined by using a best‐fit algorithm. Angular deviation, euclidean distances of the body deviation at the implant crest and apex were determined.

### 2.5 Sample Size Calculation and Statistical Analysis

A preliminary experiment with a similar setup was performed to calculate a sample size for the study. Self‐cutting implants were placed full‐guided through the template (*n* = 4) and freehand under visual control (*n* = 4) in the posterior region of acrylic models with bone density D2 and D3 after full‐guided osteotomy. The sample size was determined based on means and standard deviations using G‐Power (G‐Power 3.1.9.2., HHU Düsseldorf, Germany). A number of 40 samples were computed as necessary per group to achieve a power of 0.8 with alpha being 0.05.

For the statistical analysis, all data was sorted using an Excel spreadsheet. Statistical analyses were performed using SPSS statistical software (SPSS Statistics, version 29, IBM, Armonk, New York, United States).

Prior to statistical analysis, the dataset was assessed for a normal distribution and homoscedasticity using Q‐Q‐plots and Levene statistics (Appendix S1).

Data were analyzed using a multivariate analysis, post hoc *t*‐tests, and Bonferroni‐adjustment method. The global significance level was set at 0.05.

In anterior position, a non‐parametric test for statistical analysis was chosen. Subsequent pairwise comparisons were carried out using the Wilcoxon‐Mann–Whitney rank sum test with adjustments for repeated measures using the Bonferroni method. Again, the significance level was set at 0.05.

## 3 Results

### 3.1 Global Results

The results of the multivariate analysis showed a significant influence of bone density on coronal deviation (*p* < 0.01) and the influence of insertion protocol on angular (*p* < 0.01), coronal (*p* < 0.01) and apical deviation (*p* = 0.03). An effect of implant position on angular (*p* < 0.01) and apical (*p* < 0.01) deviation was demonstrated (Table 3).

#### 3.1.1 Effect of Bone Density and Insertion Technique on Accuracy of Implant Placement in Posterior Sites and Implant Placement in Narrow Angulated Ridges

**FIGURE 5:**
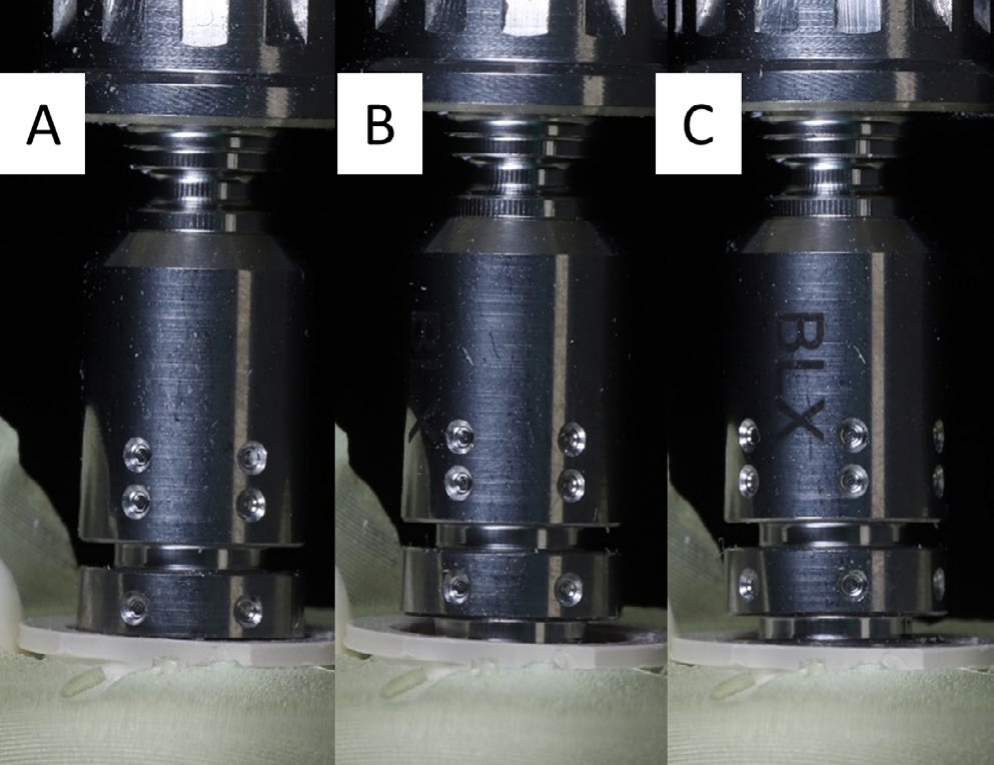
Depth mark on the insertion instrument: The width of the depth mark allows certain variation during implant insertion. The surgeon can stop the insertion at the top (A), bottom (B), or centre (C) of the mark.

The results reflect a significant influence of the insertion protocol, with significant differences particularly in soft bone (Table 3, Figure 5).

**FIGURE 6:**
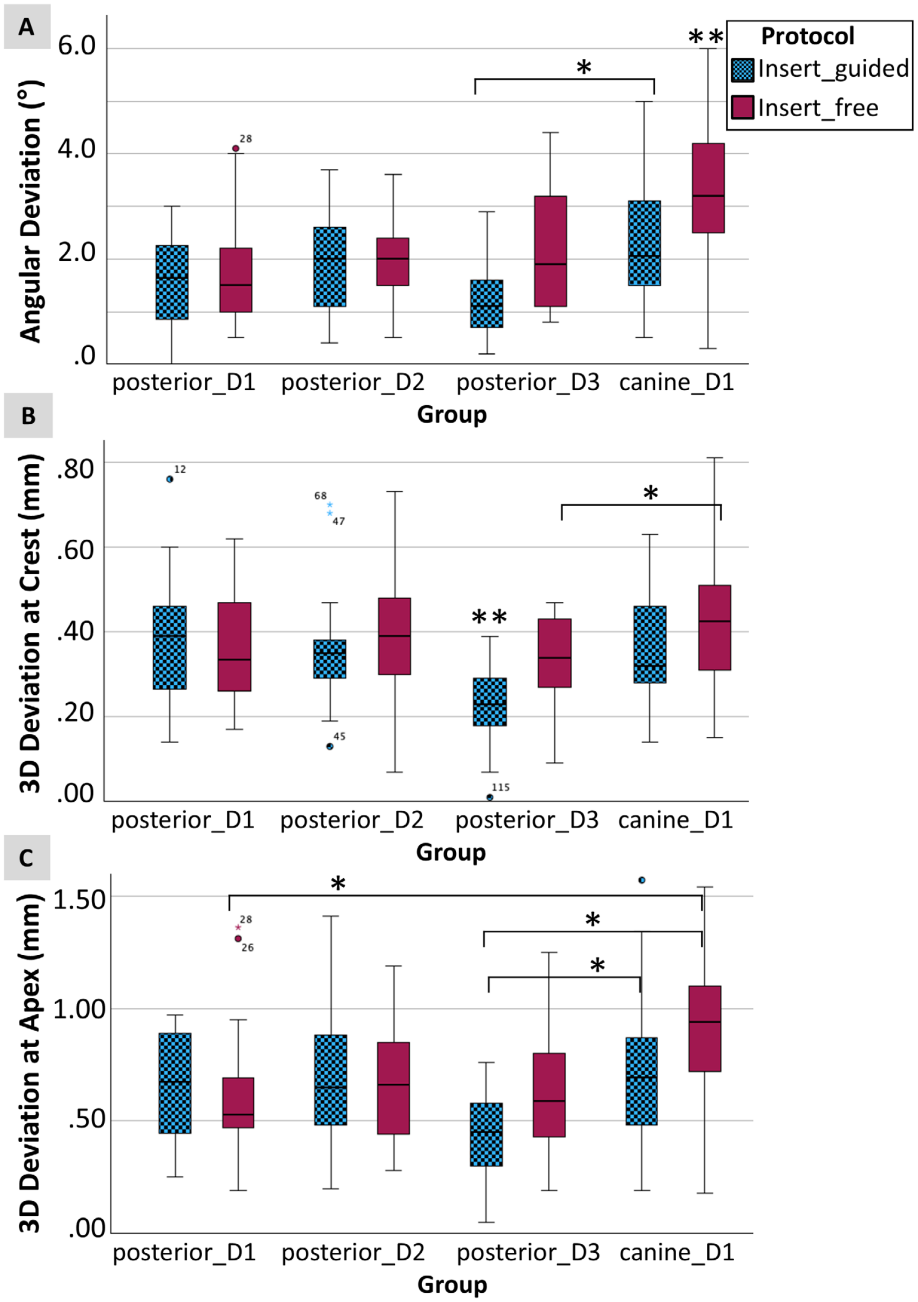
Box plots showing the angular deviation (A), 3D deviation at crest (B), and 3D deviation at apex (C) of implant placement in the posterior region with bone densities D1, D2, D3, and the canine region with bone density D1 for the protocols *Insert_guided* and *Insert_free*. *Statistically significant. **Statistically significant compared to all other groups.

The lowest mean deviations were found for guided implant placement in bone density D3 for the angle 1.2° [95% CI: 0.9–1.5], the 3D deviation at the crest 0.22 mm [95% CI 0.18–0.26] and the 3D deviation at the apex 0.44 mm [95% CI 0.35–0.53] (Table 2, Figure 6).

**TABLE 2:** Angular, crestal, and apical deviations between planned and actual achieved implant position in different positions (anterior, canine, posterior), protocols (*Insert_guided*, *Insert_free*, *Only pilot_Insert free*), and bone densities (D1, D2, D3).

Variables	Dimensions								
	Position^a^	Protocol^a^	Density^a^	*n*	Md	IQR	M	CI (95%)	SD
Angular Deviation (°)	Posterior	Insert_guided	D1	20	1.7	1.5	1.6	1.2 to 1.9	0.8
			D2	20	2.0	2.6	1.8	1.4 to 2.3	0.9
			D3	20	1.1	1.1	1.2	0.9 to 1.5	0.7
		Insert_free	D1	20	1.5	1.2	1.7	1.3 to 2.2	0.9
			D2	20	2.0	1.1	2.0	1.7 to 2.3	0.8
			D3	20	1.9	2.2	2.2	1.6 to 2.7	1.2
		Control	D1	20	1.9	1.35	1.7	1.4 to 2.1	0.7
	Canine	Insert_guided	D1	30	2.1	1.7	2.5	1.8 to 3.1	1.6
		Insert_free	D1	30	3.2	1.8	3.2	2.6 to 3.6	1.4
	Anterior	Insert_guided		14	3.4	1.9	3.5	2.8 to 4.1	1.1
		Insert_free		14	4.5	2.7	4.5	3.7 to 5.4	1.5
		Only_Pilot_ Insert_Free		14	7.1	3.3	7.3	6.2 to 8.5	2.0
		Control		14	2.7	1.2	2.6	2.1 to 3.1	0.88
3D Deviation at Crest (mm)	Posterior	Insert_guided	D1	20	0.39	0.20	0.38	0.31 to 0.45	0.15
			D2	20	0.35	0.11	0.35	0.29 to 0.41	0.13
			D3	20	0.23	0.13	0.22	0.18 to 0.26	0.09
		Insert_free	D1	20	0.33	0.22	0.34	0.29 to 0.39	0.12
			D2	20	0.39	0.21	0.38	0.31 to 0.45	0.15
			D3	20	0.34	0.17	0.33	0.28 to 0.37	0.10
		Control	D1	20	0.35	0.17	0.37	0.29 to 0.44	0.16
	Canine	Insert_guided	D1	30	0.32	0.19	0.35	0.30 to 0.40	0.13
		Insert_free	D1	30	0.42	0.21	0.41	0.36 to 0.47	0.14
	Anterior	Insert_guided		14	0.52	0.14	0.52	0.45 to 0.59	0.11
		Insert_free		14	0.83	0.23	0.85	0.76 to 0.94	0.15
		Only_Pilot_Insert_Free		14	0.47	0.26	0.47	0.35 to 0.59	0.20
		Control		14	0.60	0.52	0.73	0.55 to 0.90	0.30
3D Deviation at Apex (mm)	Posterior	Insert_guided	D1	20	0.67	0.45	0.66	0.55 to 0.76	0.23
			D2	20	0.65	0.41	0.69	0.55 to 0.84	0.32
			D3	20	0.45	0.31	0.44	0.35 to 0.53	0.19
		Insert_free	D1	20	0.53	0.22	0.60	0.46 to 0.73	0.29
			D2	20	0.66	0.43	0.67	0.55 to 0.79	0.27
			D3	20	0.59	0.41	0.64	0.51 to 0.78	0.30
		Control	D1	20	0.66	0.40	0.63	0.53 to 0.74	0.23
	Canine	Insert_guided	D1	30	0.69	0.40	0.74	0.59 to 0.88	0.38
		Insert_free	D1	30	0.94	0.40	0.90	0.78 to 1.02	0.31
	Anterior	Insert_guided		14	1.36	0.51	1.32	1.14 to 1.49	0.30
		Insert_free		14	1.95	0.60	1.88	1.67 to 2.09	0.36
		Only_Pilot_Insert_Free		14	1.92	0.65	1.94	1.74 to 2.13	0.33
		Control		14	1.32	0.77	1.29	1.06 to 1.53	0.41
Buccal Deviation at Crest (mm)	Anterior	Insert_guided		14	−0.33	0.84	−0.05	−0.30 to 0.19	0.43
		Insert_free		14	−0.73	0.60	−0.44	−0.81 to 0.08	0.62
		Only_Pilot_Insert_Free		14	0.13	0.31	0.12	0.01 to 0.25	0.21
		Control		14	0.41	0.17	0.34	0.22 to 0.47	0.22
Buccal Deviation at Apex (mm)	Anterior	Insert_guided		14	−0.90	2.16	−0.23	−0.92 to 0.45	1.19
		Insert_free		14	−1.77	0.68	−1.31	−2.04 to 0.57	1.26
		Only_Pilot_Insert_Free		14	1.69	3.32	0.89	−0.07 to 1.85	1.67
		Control		14	0.85	0.50	0.79	0.49 to 1.09	0.52

Abbreviations: CI, confidence interval; IQR, interquartile range; M, mean; Md, median; N, sample size; SD, standard deviation.

^a^ Nominal data.

The maximal deviations are mentioned as they are clinically relevant for the assessment of necessary safety distances: These were found in bone density D1 with angle deviation of 4.0° (*Insert_free*), 3D deviation at the crest of 0.76 mm (*Insert_guided*), and 3D deviation at the apex of 1.41 mm (*Insert_free*).

#### 3.1.2 Effect of Insertion Technique on Accuracy of Implant Placement in Narrow Angulated Ridges

The lowest mean deviations were found for *Insert_guided* implant placement for the angle 2.5° [95% CI: 1.8–3.1], the 3D deviation at the crest 0.35 mm [95% CI 0.30–0.40] and the 3D deviation at the apex 0.74 mm [95% CI 0.59–0.88] (Table 2, Figure 6).

The maximal deviations were in the *Insert_free* group with angle deviation of 6.8°, 3D deviation at the crest of 0.81 mm, and 3D deviation at the apex of 1.78 mm.

In comparison with implant accuracy in posterior sites, the deviations observed in narrow angulated ridges were found to be significantly higher for angle and 3D deviation at the apex in both *Insert_guided* and *Insert_free* (*p* < 0.01) (Table 3).

**TABLE 3:** Results of multivariate analysis showing effects of protocol, density, position and their combination on angular deviation (°), 3D deviation at crest (mm), and 3D deviation at apex (mm).

Variable	Factor	df	*F*‐value	*p*	Cohens *f*
Angular Deviation (°)	Protocol Density Position	1	9.982	0.002*	0.2366
		2	0.791	0.455	0.0953
		1	25.358	< 0.001*	0.3779
	Interaction Protocol*Density Interaction Protocol*Position	2	1.848	0.161	0.1429
		2	1.544	0.216	0.0953
3D Deviation at Crest (mm)	Protocol Density Position	1	8.066	0.005*	0.2119
		2	6.882	< 0.001*	0.2785
		1	0.304	0.582	0.0447
	Interaction Protocol*Density Interaction Protocol*Position	2	2.837	0.029*	0.1788
		2	3.068	0.082	0.1315
3D Deviation at Apex (mm)	Protocol Density Position	1	4.924	0.028*	0.1665
		2	2.240	0.109	0.1601
		1	10.284	0.002*	0.2412
	Interaction Protocol*Density	2	2.320	0.101	0.1601
	Interaction Protocol*Position	2	3.447	0.065	0.1391

* Statistically Significant.

#### 3.1.3 Effect of Drilling Protocol and Insertion Technique on Accuracy of Implant Placement in Anterior Sites

**FIGURE 7:**
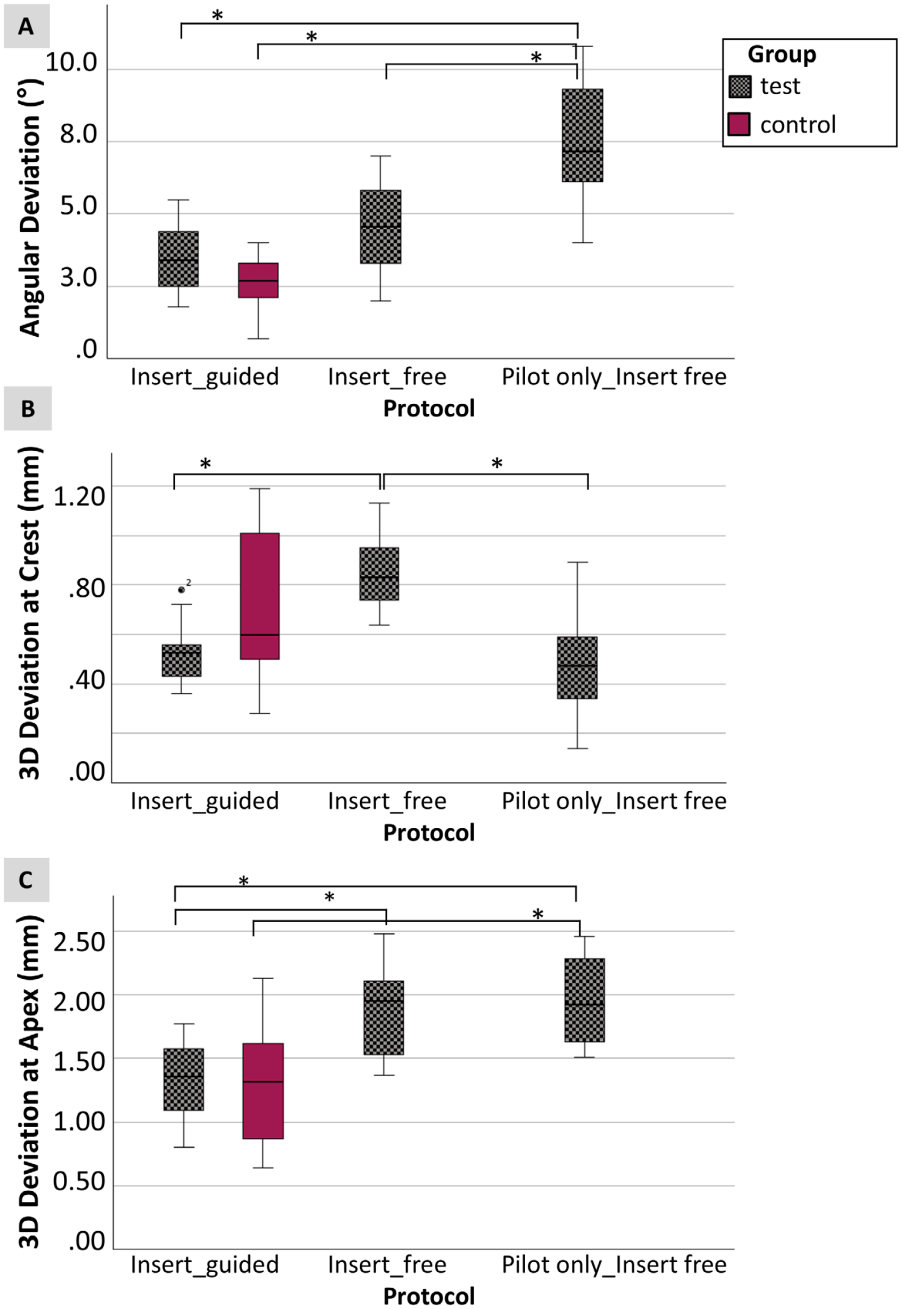
Box plots showing the angular deviation (A), 3D deviation at crest (B) and 3D deviation at apex (C) of implant placement in the anterior region (test and control) for the protocols *Insert_guided*, *Insert_free*, *Pilot only_Insert free*. *Statistically significant.

Significant differences between the accuracy of the different protocols were observed for anterior implant placement (Table 4, Figure 7).

**TABLE 4:** Results of mean value comparisons of implant placement in anterior sites with different guide protocols for mean angular (°), 3D deviation at the crest (mm), and 3D deviation at the apex (mm) with Wilcoxon‐Mann–Whitney rank sum test and adjusted significance level to *p* < 0.01 (bonferroni correction).

Variable	A	B	*p*
Angular Deviation (°)	Insert_guided	Insert_free	0.04
	Insert_guided	Pilot only_Insert free	< 0.01*
	Insert_guided	Control	0.03
	Insert_free	Pilot only_Insert free	< 0.01*
	Pilot only_Insert free	Control	< 0.01*
3D Deviation at Crest (mm)	Insert_guided	Insert_free	< 0.01*
	Insert_guided	Pilot only_Insert free	0.54
	Insert_guided	Control	0.05
	Insert_free	Pilot only_Insert free	< 0.01*
	Pilot only_Insert free	Control	0.031
3D Deviation at Apex (mm)	Insert_guided	Insert_free	< 0.01*
	Insert_guided	Pilot only_Insert free	< 0.01*
	Insert_guided	Control	0.24
	Insert_free	Pilot only_Insert free	0.60
	Pilot only_Insert free	Control	< 0.01*

* Statistically Significant.

For the 3D deviations at the crest, the lowest mean deviations were found in the *Pilot only_Insert free* group with 0.47 mm [95% CI 0.35–0.59]. For the angle and 3D deviation at the apex, the lowest mean deviations were measured in the *Insert_guided* group with 3.5° [95% CI 2.8–4.1] and 1.32 mm [95% CI 1.14–1.49] (Table 2, Figure 7).

The maximal deviations were found in the *Pilot only_Insert free* group with an angle deviation of 10.8° and a 3D deviation at the apex of 2.62 mm and in the *Insert_free* group with a 3D deviation at the crest of 1.13 mm.

In terms of the buccal deviation, vestibular deviations were mainly observed in the *Pilot only_Insert free* group, whereas palatal deviations were more common in the *Insert_free* groups (Table 2).

## 4 Discussion

The results of the present in vitro study indicate that both the bone constitution (bone density, bone shape, extraction sockets) and the insertion technique influence implant accuracy when using self‐cutting implants. Especially in soft bone, implant accuracy benefited from fully guided insertion. In regions with less visual orientation of the implant axis, for example, due to a strongly deviating axis of neighboring teeth, there were generally higher deviations observed, which could also be minimized by a guided insertion. For immediate implant placement in the anterior region, a direct view of the surgical field proved advantageous for positioning the implant shoulder at the expense of the accuracy of implant angulation and apex offset.

Despite the in vitro conditions and standardization of many factors, variations from the planned to the executed position were still observed in all groups. Several sources of error may be involved. During data acquisition, deviations may occur when generating 3D surface data and acquiring CBCT data (Edelmann et al. 2021; Tahmaseb et al. 2018; Wei et al. 2021; Wismeijer et al. 2018). The type and dimensions of the surgical template (El Kholy, Ebenezer, et al. 2019; Lin, Ishikawa, et al. 2020; Lin, Wu, et al. 2020; Pozzi et al. 2016; Raico Gallardo et al. 2017) and the design of the sleeve (height, position) and offset parameters (El Kholy, Janner, et al. 2019; Guentsch et al. 2022; Kessler et al. 2021; Lim et al. 2021; Tallarico et al. 2019; Ye et al. 2019) may influence the outcome and were set consistently in our study. Although there are several manufacturing processes available for the production of the surgical template, different printing technologies were found not to be significant in influencing the final implant accuracy (Herschdorfer et al. 2021). As slight variations can occur due to differences in print layer thickness and print orientation between additive printing technologies (Al‐Dulaijan et al. 2023; Farkas et al. 2023), we followed the manufacturer's precise instructions in our study.

Deviations may also be caused by the insertion instrument. To avoid friction, a small amount of clearance should be maintained between the components. The width of the depth marker further allows the surgeon to stop the insertion at the top, bottom, or center of the mark, which directly influences the assessed accuracy (Figure 5). Furthermore, when trying to achieve a final positioning of the implant index, slight deviations arise as an additional factor. This can lead to variations in the depth, as has been seen in previous studies. In a randomized clinical trial, Waltenberger et al. (2024) assessed implant accuracy with regard to the desired depth and index. The study demonstrated a mean global depth variation of 0.27 mm in cases of fully guided implant placement of single‐tooth implants. These results were consistent with the findings of other studies that had optimal support for the template. This global error occurred seemingly due to the consistent offset of 0.3 mm in the template design process on the one hand, or variations in where to place the depth marker on the other hand. In contrast, with a direct view of the surgical field and a clear reference for the position of the implant shoulder, coronal accuracy can be improved. However, adjusting the coronal implant position directly to the clinical bone situation in direct vision may also result in greater deviations compared to the planned position. In our study, the buccal bone lamella served as a depth marker stop during immediate implant placement in the anterior region.

For late implant placement, the present study showed the smallest deviations in soft bone. This appeared to be due to the fact that the residual bone offers less resistance after osteotomy, allowing the implant to reach the target position more passively. Lateral guidance of the sleeve may be transferred more directly to the implant. The relationship between bone density, primary stability, and insertion technique was investigated early by Bayarchimeg et al. (2013), without discussing the possible influence of these factors on accuracy. In their study, Chen et al. (2024) found an influence of bone density on the accuracy of guided implant placement. In the present study, this is now additionally related to the different possible drilling protocols and the different implant geometries.

The findings indicated a multifactorial interaction of diverse influencing factors, which can ultimately lead to deviations in vitro, as has been observed in comparable studies. El Kholy, Ebenezer, et al. (2019) showed 3D coronal deviations up to 0.81 mm, apical up to 1.17 mm, and angular up to 3.90° with comparable guided implant placement of a conical‐parallel implant in hard bone in vitro.

Especially in the anterior region, precise implant positioning is a prerequisite for successful implant placement with contemporary loading protocols. The space and bone available are often highly limited. The buccal lamella has to be protected to avoid complications, for example, soft and hard tissue dehiscence (Hamilton et al. 2023; Mao et al. 2021; Mazzotti et al. 2018; Nisapakultorn et al. 2010) and to achieve a predictable aesthetic outcome. Therefore, an additional evaluation of the implant position in the bucco‐oral direction was performed for the anterior region in this study. The results indicate the smallest deviations in the *Insert_guided* group. The *Insert_free* group tended to deviate palatally, while the *Pilot only_Insert free* group tended to deviate buccally for apical and coronal position (Table 4). For immediate implant placement, the diameter of the socket is an important factor. Implants were inserted into the palatal bone after osteotomy without a complete osseous socket. This results in greater palatal resistance during implant placement and subsequently more frequent buccal misalignment of the implant. Placement without a template allows the surgeon to counteract this phenomenon under visual control. If only a pilot osteotomy is done fully guided, the following drills experience further palatal resistance resulting in greater apical deviation. This may be a reason for the greater buccal deviations in the *Pilot only_Insert free* group. The surgeon should be aware of the different sources of influence and the advantages and disadvantages of each insertion protocol aiming to minimize inaccuracies. These findings can be transferred to the premolar region, as the geometry of the alveolus is usually similar and the oral bone wall also serves as guidance for implant positioning. However, in the molar region, immediate implants are planned to be placed intraseptally, which renders the results inappropriate for generalization to this region.

Extrapolation of results to the mandible must be viewed critically, as both bone density and anatomy often differ from the maxilla. The canine region is the most suitable for comparison, as the model has a narrow alveolar ridge in this region; the neighbouring teeth provide little guidance, and the bone is very dense.

In relation to implant geometry, the self‐cutting implants demonstrated efficacy even in simulated critical scenarios. The gripping of the thread flanks was advantageous for secure positioning. These findings are consistent with the in vitro study of El Kholy, Ebenezer, et al. (2019), who observed a significant impact of implant geometry on implant position accuracy. The study revealed that conical implants (Bone Level Tapered, Straumann AG) exhibited superior accuracy in comparison to parallel‐walled macro designs (Bone Level, Straumann AG), particularly in scenarios involving hard bone and fully guided implant placement. This discrepancy in performance was attributed to the varying resistances encountered during implant placement. The Bone Level X implant utilized in this study bears a resemblance to the BLT implant with regard to the anatomy of the implant body. However, it possesses an even more tapered thread profile and more pronounced thread flanks. In the context of soft bone, it is anticipated that the insertion step will exert a greater influence on the implant position, a phenomenon attributable to the aggressive threaded implant geometry and the low resistance of the bone. In hard bone, the pre‐drilled hole appears to determine the path of the implant. However, in cases where there is less reference due to the position of the adjacent teeth (alveolar ridge), the accuracy of insertion through the template also appears to benefit in hard bone.

Further studies should clarify whether variations in final implant position originate due to the osteotomy of the implant site, or due to the subsequent placement. The development of a study protocol to visualize the implant drilling process for evaluation purposes is recommended. The testing of different implant geometries under various conditions is also advised. The evaluation of an ideal protocol and implant anatomy for diverse clinical scenarios would provide clarity regarding these matters.

Critical consideration should be given to the evaluation strategy for the accuracy of the implant position with coDiagnostiX software algorithm. Despite the evaluation tool's extensive utilization in numerous studies, the precise algorithm remains undisclosed. As Herstell et al. (2022) showed, no significant differences were found when several evaluation strategies were compared. Based on these results, the tool was chosen to analyze the implant position.

As a limiting factor, the small sample size and the constraints of independence of the study should be discussed. Moreover, the apical deviations in particular should be given due consideration. The observed variations between the groups can be attributed to the differences in implant length alone. Angular deviations are more pronounced in the case of longer implants with regard to apical accuracy.

Due to the fact that the study was conducted in vitro to exclude external influences that may have an additional impact on the accuracy of implant placement, greater variation may be expected in the clinical situation, which also can be considered a limitation in transferability to the clinic. In their review and meta‐analysis of randomized controlled clinical trials, Tattan et al. (2020) show 3D coronal deviations up to 3.35 mm, 3D apical deviations up to 3.64 mm, and angular deviations of up to 6.82° with fully guided implantation. With a similar planning workflow, surgical templates, and implant osteotomy to the present experiment, a clinical study from our department found 3D coronal deviations of 0.55 mm (0.16–1.09), 3D apical deviations of 0.91 mm (0.15–2.09), and angular deviations of 3.06° (0.2–12.8) (Waltenberger et al. 2024). In their prospective clinical study, Schnutenhaus et al. (2024) showed similar results with deviations of 0.87 mm (0.61–1.13), 3D apical deviations of 1.01 mm (0.73–1.29), and angular deviations of 3.2° (2.5–3.8). Therefore, we can provide a factor of 35% increase in variation when transferring from in vitro to in vivo conditions in this special example. In our scenario, influences such as reduced visibility due to saliva and blood, limited space due to restricted mouth opening, and patient movement during implant drilling and placement were excluded. Precise reference points (clear view of the alveolus or flat bone level) would be unrealistic in a clinical situation. Water cooling during implant drilling was also not used. In addition, the influence of flap preparation or closed procedure (Behneke et al. 2012) was excluded by using models without soft tissue simulation. By standardizing the experimental design and eliminating factors that are known to influence the outcome, it was possible to focus on the research question, which could form the basis for future research projects. The study justifies further clinical investigation of sCAIS in critical situations (soft bone and extraction sockets) as the factor of bone quality and degree of guidance do play a vital and not fully investigated role in achieving a predictable implant accuracy.

## 5 Conclusion

Based on the findings of this in vitro study with its limitations, it can be concluded that as follows:

- Full guided osteotomy and fully guided insertion increase the accuracy compared to full‐guided osteotomy and freehand insertion;
- The bone quality does influence the implant accuracy significantly, with hard bone as a factor for an increase in deviation;
- In immediate anterior extraction sockets, freehand placement may enhance the precise positioning of the implant shoulder. The apical and angular accuracy is best with fully guided insertion.

## Author Contributions

**Caroline Streichfuss:** conceptualization, data curation, formal analysis, investigation, methodology, project administration, visualization, writing – original draft. **Stefan Wolfart:** conceptualization, formal analysis, investigation, methodology, resources, project administration, supervision, validation, visualization, writing – review and editing. **Lukas Waltenberger:** writing – review and editing, conceptualization, formal analysis, investigation, methodology, project administration, resources, supervision, validation, visualization.

## Ethics Statement

The authors have nothing to report.

## Conflicts of Interest

C.S. declares no conflicts of interest. S.W. reports Grants, Personal fees and non‐financial support from Oral Reconstruction Foundation, ITI, Straumann, DGI; personal fees from Geistlich Biomaterials, Camlog, Quintessence Publishing, APW, DGÄZ, University of RWTH Aachen, Greifswald and Frankfurt; non‐financial support from Ivoclar Vivadent, grants from Deutsche Forschungsgemeinschaft (German research foundation), DGPro, DGI outside the submitted work. L.W. reports a grant from ITI and AG Start (RWTH Aachen University) as well as personal fees from DGI and Camlog.

## Supporting information


Appendix S1.

## Data Availability

The data that support the findings of this study are available from the corresponding author upon reasonable request.
