# Supplementary material for: Influence of Bone Density and Guide Protocol on the Accuracy of Self‐Cutting Implants Using Static Guided Implant Placement—An In Vitro Study
Source: Clin Oral Implants Res. 2025 Jul 3;36(10):1248–60. doi: 10.1111/clr.14470 (PMC12491919; doi:10.1111/clr.14470)
Supplement: Supplementary file 1 — Appendix S1. [file CLR-36-1248-s001.zip › clr14470-sup-0001-Supinfo1@Supplementary Material_05.05.2025.docx]

# **Supplementary Material**

A graphical analysis of the Q-Q plots for the different implant positions was performed to test for normal distribution. In addition, standardised plots were generated to test the data for homoscedasticity.


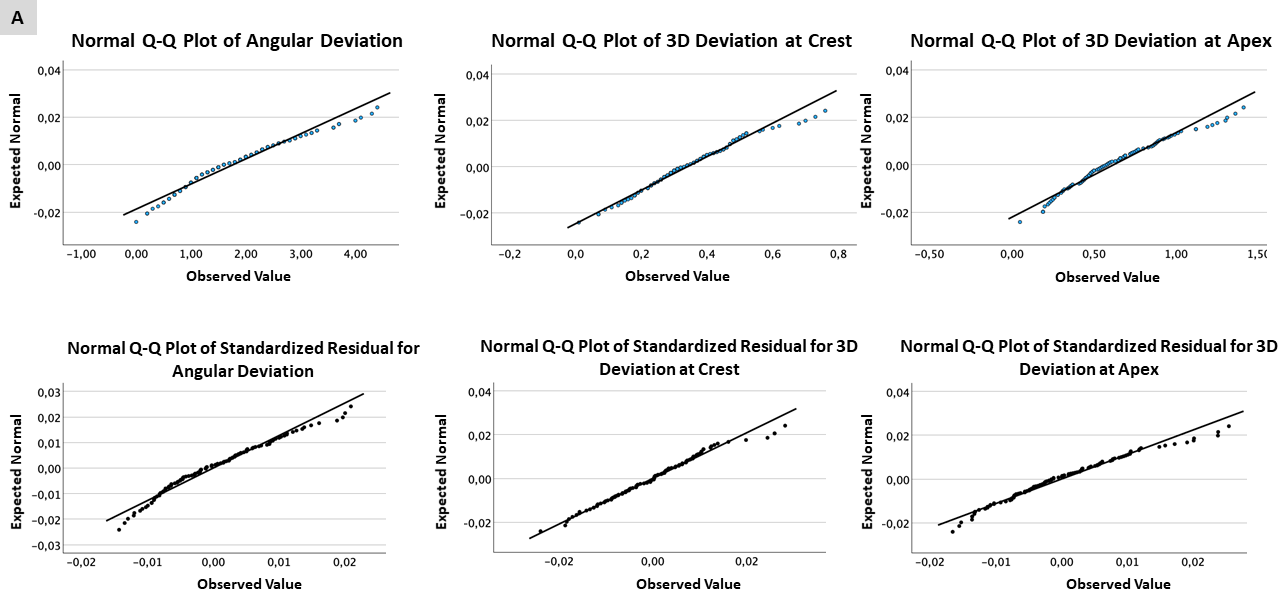
Figure 8: Normal Quantile-Quantile plot showing values for angular deviation, 3D deviation at crest and 3D deviation at apex in posterior position assessing normality graphically. Normal Quantile-Quantile plots of standardised residual for angular deviation, 3D deviation at crest and 3D deviation at apex to test homoscedasticity. A: poster
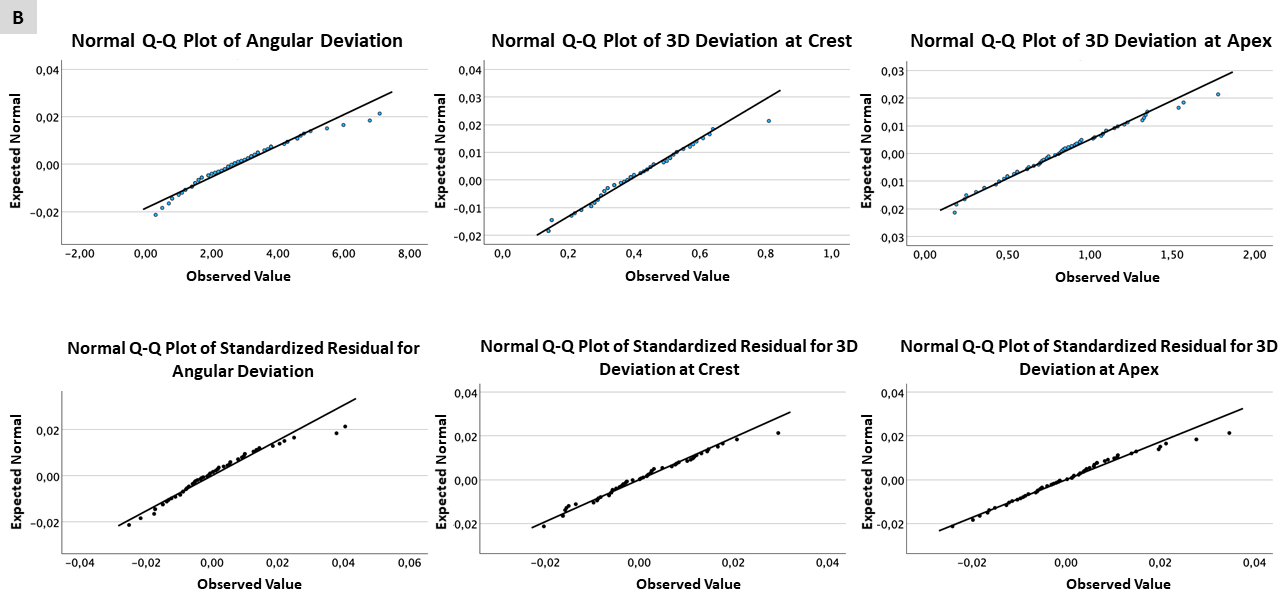
ior position. B: anterior position.

A levene test was performed to evaluate homogeneity of variances.

| **Variable** |  | **Levene Statistics** | **df1** | **df2** | **Sig.** |
| --- | --- | --- | --- | --- | --- |
| **Angular Deviation (°)** | Based on median | 2.048 | 7 | 178 | .052 |
| **3D Deviation at Crest (mm)** | Based on median | .932 | 7 | 178 | .483 |
| **3D Deviation at Apex (mm)** | Based on median | .989 | 7 | 178 | .441 |

Tab 6: Results of Levene-test for evaluation of homogeneity of variances.
