# Supplementary figures and images for: Influence of Bone Density and Guide Protocol on the Accuracy of Self‐Cutting Implants Using Static Guided Implant Placement—An In Vitro Study
Source: Clin Oral Implants Res. 2025 Jul 3;36(10):1248–60. doi: 10.1111/clr.14470 (PMC12491919; doi:10.1111/clr.14470)

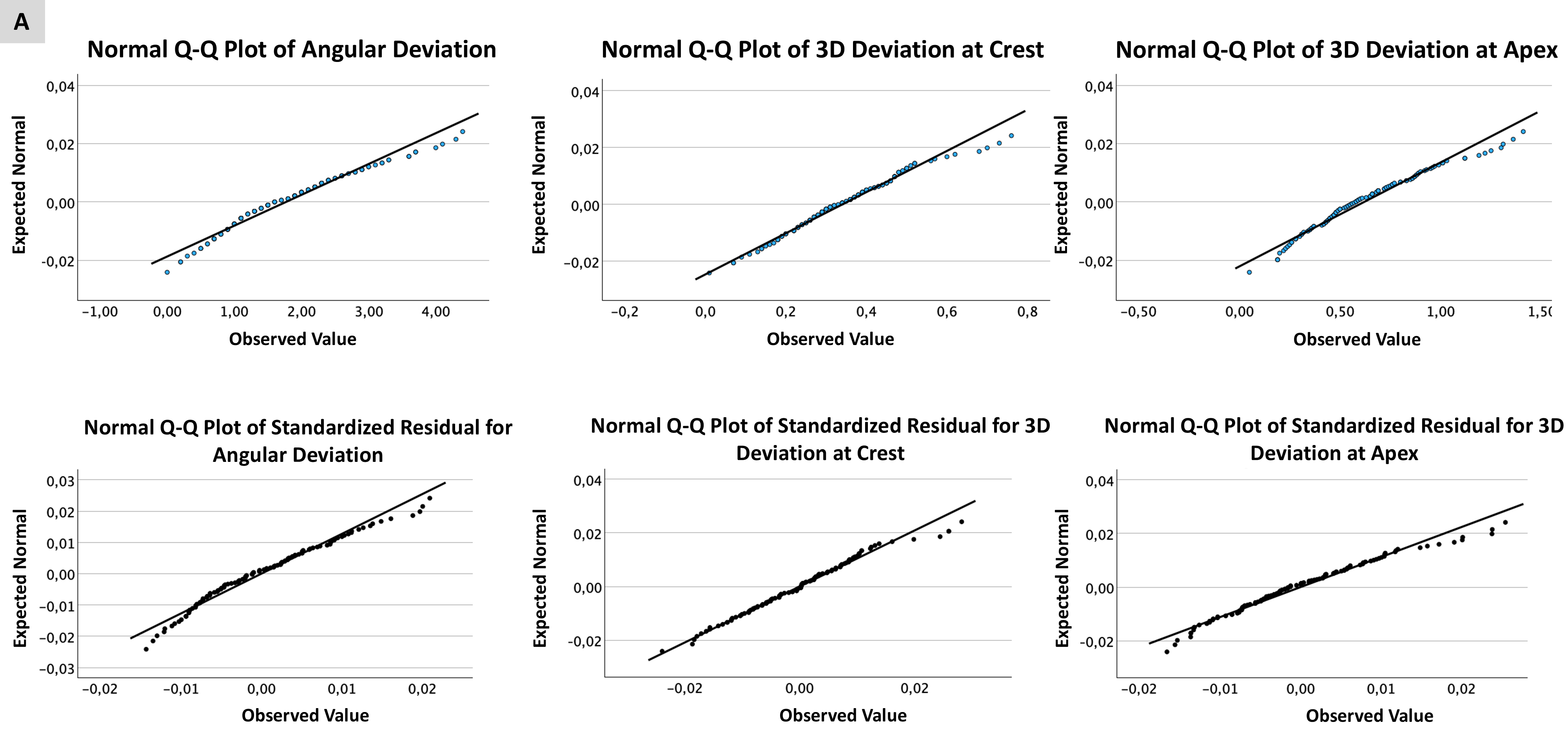

Supplement: Supplementary file 1 — Appendix S1. [file CLR-36-1248-s001.zip › CLR_14470_f8_Figure_8A.PNG]

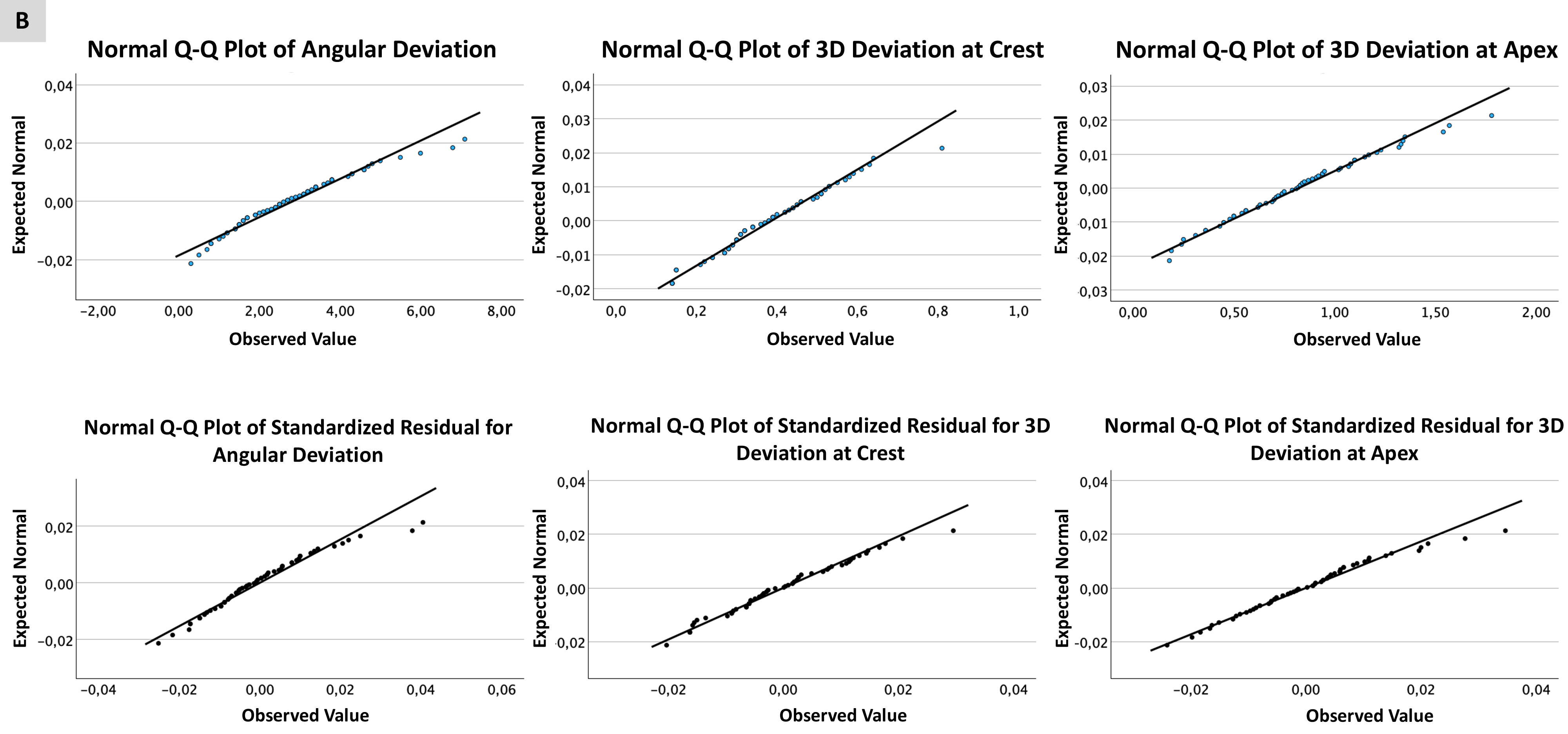

Supplement: Supplementary file 1 — Appendix S1. [file CLR-36-1248-s001.zip › CLR_14470_f8_Figure_8B.PNG]
